# Supplementary material for: Biological insights from plasma proteomics of non-small cell lung cancer patients treated with immunotherapy
Source: Front Immunol. 2024 Feb 29;15:1364473. doi: 10.3389/fimmu.2024.1364473 (PMC10937428; doi:10.3389/fimmu.2024.1364473)
Supplement: Supplementary file 1 [file DataSheet_1.pdf]

Supplemental data online

Supplemental Tables

Table S1: Treatment modalities used in the NSCLC patient cohort.

Treatment modality	Drugs	Number of patients
ICI	Pembrolizumab	69
	Nivolumab	26
	Atezolizumab	4
	Durvalumab	1
ICI + Chemo	Pembrolizumab + Carboplatin + Pemetrexed	83
	Pembrolizumab + Carboplatin + Paclitaxel	27
	Pembrolizumab + Cisplatin + Pemetrexed	9
	Atezolizumab + Bevacizumab + Carboplatin + Paclitaxel	2
	Pembrolizumab + Nab-Paclitaxel + Carboplatin	1
	Durvalumab + Carboplatin +Paclitaxel	1
	Nivolumab, Carboplatin, Paclitaxel	1
	Nivolumab, Carboplatin, Pemetrexed	1

ICI, PD-1/PD-L1 inhibitors; ICI+Chemo, PD-1/PD-L1 inhibitors combined with chemotherapy.

Table S2: Chemotherapy-treated NSCLC cohort - demographics, clinicopathological characteristics and outcomes.

Metric	Overall
**n**	20
**Age, mean (SD)**	65.2 (6.4)
**ECOG, n (%)**	
0	11 (55.0)
1	7 (35.0)
2	1 (5.0)
3	1 (5.0)
**Sex, n (%)**	
Female	6 (30.0)
Male	14 (70.0)
**Site, n (%)**	
Thoraxklinik DE	20 (100.0)
**Line of treatment, n (%)**	
Advanced	16 (80.0)
Unknown	4 (20.0)
**Tumor PD-L1 Level, n (%)**	
High (≥50%)	5 (25.0)
Low (1%-49%)	10 (50.0)
Negative (<1%)	5 (25.0)
**Histology, n (%)**	
Adenocarcinoma	11 (55.0)
Squamous cell lung cancer	8 (40.0)
Other	1 (5.0)
**RECIST criteria, n (%)**	
CR, PR, SD	6 (30.0)
PD	12 (60.0)
NA	2 (10.0)

CR, complete response; NA, not applicable; PD, progressive disease; PR, partial response; SD, stable disease.

Table S3: Melanoma cohort - demographics, clinicopathological characteristics and outcomes.

Metric	Overall
**n**	20
**Age, mean (SD)**	65.5 (14.3)
**ECOG, n (%)**	
0	17 (85.0)
1	1 (5.0)
Not reported	2 (10.0)
**Sex, n (%)**	
Female	9 (45.0)
Male	11 (55.0)
**Site, n (%)**	
Hadassa IL	16 (80.0)
Haemek IL	3 (15.0)
Bnai-Zion IL	1 (5.0)
**Line of treatment, n (%)**	
First	13 (65.0)
Advanced	3 (15.0)
Unknown	4 (20.0)
**Histology, n (%)**	
Cutaneous Melanoma	10 (50.0)
Uveal Melanoma	4 (20.0)
Mucosal Melanoma	2 (10.0)
Acral Melanoma	1 (5.0)
Other/NA	3 (15.0)
**RECIST criteria, n (%)**	
CR, PR, SD	9 (45.0)
PD	9 (45.0)
NA	2 (10.0)

CR, complete response; NA, not applicable; PD, progressive disease; PR, partial response; SD, stable disease.

Table S4: Associations between sPD-1 and clinical parameters.

Metric	Overall	Low sPD-1	High sPD-1	P-value (FDR)
**n**	225	113	112	
**Age, mean (SD)**	66.4 (8.2)	66.0 (8.4)	66.9 (7.9)	0.365 (0.657)
**ECOG, n (%)**				
0	75 (33.3)	37 (32.7)	38 (33.9)	0.548 (0.705)
1	128 (56.9)	64 (56.6)	64 (57.1)	
2	19 (8.4)	10 (8.8)	9 (8.0)	
3	2 (0.9)	2 (1.8)		
Not reported	1 (0.4)		1 (0.9)	
**Sex, n (%)**				
Female	84 (37.3)	33 (29.2)	51 (45.5)	0.017 (0.051)
Male	141 (62.7)	80 (70.8)	61 (54.5)	
**Site, n (%)**				
Thoraxklinik DE	98 (43.6)	40 (35.4)	58 (51.8)	0.061 (0.137)
Sheba IL	60 (26.7)	38 (33.6)	22 (19.6)	
Asklepios DE	16 (7.1)	10 (8.8)	6 (5.4)	
Rabin IL	10 (4.4)	4 (3.5)	6 (5.4)	
Other	41 (18.2)	21 (18.6)	20 (17.9)	
**Line of treatment, n (%)**				
First	165 (73.3)	84 (74.3)	81 (72.3)	0.849 (0.849)
Advanced	60 (26.7)	29 (25.7)	31 (27.7)	
**Treatment, n (%)**				
ICI	100 (44.4)	38 (33.6)	62 (55.4)	0.002 (0.009)
ICI + Chemo	125 (55.6)	75 (66.4)	50 (44.6)	
**Tumor PD-L1 Level, n (%)**				
High (≥50%)	89 (39.6)	32 (28.3)	57 (50.9)	0.001 (0.009)
Low (1%-49%)	53 (23.6)	31 (27.4)	22 (19.6)	
Negative (>1%)	60 (26.7)	41 (36.3)	19 (17.0)	
Unknown	23 (10.2)	9 (8.0)	14 (12.5)	
**Histology, n (%)**				
Adenocarcinoma	156 (69.3)	81 (71.7)	75 (67.0)	0.519 (0.705)
Squamous Cell Carcinoma	46 (20.4)	23 (20.4)	23 (20.5)	
Other	23 (10.2)	9 (8.0)	14 (12.5)	
**RECIST criteria, n (%)**				
CR, PR, SD	175 (78.4)	86 (76.7)	89 (80.1)	0.650 (0.731)
PD	48 (21.5)	26 (23.2)	22 (19.8)	
NA	2 (0.1)	1 (0.1)	1 (0.1)	

NSCLC ICI and ICI+Chemo cohorts were stratified by high and low levels of sPD-1 (9227-15 aptamer). CR, complete response; ICI, PD-1/PD-L1 inhibitors; ICI+Chemo, PD-1/PD-L1 inhibitors combined with chemotherapy; NA, not applicable; PD, progressive disease; PR, partial response; SD, stable disease.

Table S5: Top biological processes enriched in ICI monotherapy cohort.

	Source	Term name	Term id	-log10 (adjusted p value)
1	GO:BP	immune system process	GO:0002376	21.58426
2	GO:BP	adaptive immune response	GO:0002250	17.88416
3	GO:BP	immune response	GO:0006955	17.28246
4	GO:BP	positive regulation of immune system process	GO:0002684	14.00563
5	GO:BP	regulation of immune system process	GO:0002682	13.29835
6	GO:BP	lymphocyte activation	GO:0046649	13.10164
7	GO:BP	lymphocyte mediated immunity	GO:0002449	13.10164
8	GO:BP	adaptive immune response based on somatic recombination of immune receptors built from immunoglobulin superfamily domains	GO:0002460	12.66585
9	GO:BP	cell killing	GO:0001906	12.58107
10	GO:BP	leukocyte activation	GO:0045321	12.35538
11	GO:BP	cell activation	GO:0001775	12.30476
12	GO:BP	T cell activation	GO:0042110	11.7129
13	GO:BP	leukocyte cell-cell adhesion	GO:0007159	11.0257
14	GO:BP	regulation of cell killing	GO:0031341	10.94615
15	GO:BP	leukocyte mediated immunity	GO:0002443	10.82761
16	GO:BP	immune effector process	GO:0002252	10.43794
17	GO:BP	regulation of lymphocyte mediated immunity	GO:0002706	10.43794
18	GO:BP	defense response	GO:0006952	10.31962
19	GO:CC	external side of plasma membrane	GO:0009897	14.23286
20	GO:CC	cell surface	GO:0009986	14.23286
21	GO:CC	plasma membrane	GO:0005886	13.42386
22	GO:CC	cell periphery	GO:0071944	11.91339
23	GO:CC	side of membrane	GO:0098552	11.77873
24	REAC	Immunoregulatory interactions between a Lymphoid and a non-Lymphoid cell	REAC:R-HSA-198933	21.89671
25	REAC	Immune System	REAC:R-HSA-168256	18.69919
26	REAC	Adaptive Immune System	REAC:R-HSA-1280218	14.75221

Table S6: Top biological processes enriched in non-responders.

	Source	Term name	Term id	-log10 (adjusted p value)
1	HPA	lung; alveolar cells type II	HPA:0301371	2.463387355
2	HPA	lung; alveolar cells type I	HPA:0301361	2.463387355
3	KEGG	Hippo signaling pathway - multiple species	KEGG:04392	3.959052061
4	REAC	RUNX3 regulates YAP1-mediated transcription	REAC:R-HSA-8951671	3.964585372
5	REAC	YAP1- and WWTR1 (TAZ)-stimulated gene expression	REAC:R-HSA-2032785	3.724222096
6	GO:CC	nucleus	GO:0005634	2.168533572

Table S7: Associations between alveolar proteins and clinical parameters

Metric	Overall	Low Alv	High Alv	P-value (FDR)
**n**	100	50	50	
**Age, mean (SD)**	67.6 (8.2)	67.9 (8.0)	67.2 (8.4)	0.679 (0.821)
**ECOG, n (%)**				
0	37 (37.0)	16 (32.0)	21 (42.0)	0.718 (0.821)
1	53 (53.0)	28 (56.0)	25 (50.0)	
2	8 (8.0)	5 (10.0)	3 (6.0)	
3	2 (2.0)	1 (2.0)	1 (2.0)	
**Sex, n (%)**				
Female	43 (43.0)	20 (40.0)	23 (46.0)	0.686 (0.821)
Male	57 (57.0)	30 (60.0)	27 (54.0)	
**Site, n (%)**				
Thoraxklinik DE	48 (48.0)	26 (52.0)	22 (44.0)	0.91 (0.910)
Sheba IL	27 (27.0)	12 (24.0)	15 (30.0)	
Asklepios DE	3 (3.0)	1 (2.0)	2 (4.0)	
Rabin IL	2 (2.0)	1 (2.0)	1 (2.0)	
Other	20 (20.0)	10 (20.0)	10 (20.0)	
**Line of treatment, n (%)**				
First	53 (53.0)	29 (58.0)	24 (48.0)	0.423 (0.821)
Advanced	47 (47.0)	21 (42.0)	26 (52.0)	
**Treatment, n (%)**				
ICI	100 (100.0)	50 (100.0)	50 (100.0)	
**Tumor PD-L1 Level, n (%)**				
High (≥50%)	65 (65.0)	35 (70.0)	30 (60.0)	0.093 (0.372)
Low (1%-49%)	13 (13.0)	8 (16.0)	5 (10.0)	
Negative (>1%)	12 (12.0)	2 (4.0)	10 (20.0)	
Unknown	10 (10.0)	5 (10.0)	5 (10.0)	
**Histology, n (%)**				
Adenocarcinoma	68 (68.0)	32 (64.0)	36 (72.0)	0.502 (0.821)
Squamous Cell Carcinoma	25 (25.0)	15 (30.0)	10 (20.0)	
Other	7 (7.0)	3 (6.0)	4 (8.0)	
**RECIST criteria, n (%)**				
CR, PR, SD	71 (71.0)	46 (92.0)	25 (50.0)	1.68E-5 (1.34E-4)
PD	28 (28.0)	4 (8.0)	24 (48.0)	
NA	1 (1.0)	0 (0.0)	1 (2.0)	

NSCLC ICI monotherapy cohort stratified by high and low levels of alveolar proteins (median of z-score normalization). Alv, alveolar cells; CR, complete response; ICI, PD-1/PD-L1 inhibitors; NA, not applicable; PD, progressive disease; PR, partial response; SD, stable disease.

Supplemental Figures

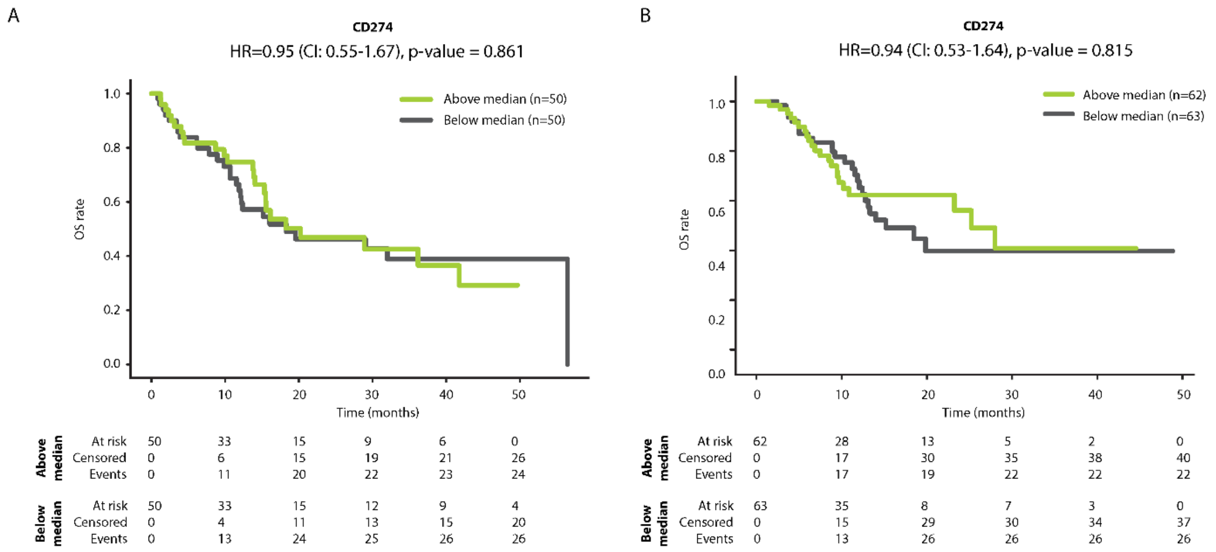

Figure S1: Kaplan-Meier plots showing the relationship between sPD-L1 fold change and overall survival. Patients were treated with PD-1/PD-L1 inhibitor monotherapy (n=100; ICI; A) or PD-1/PD-L1 inhibitors in combination with chemotherapy (n=125; ICI+chemo; B). Patients were classified as having high or low sPD-L1 fold change (sPD-L1-high or sPD-L1-low, respectively) using the median sPD-L1 fold change as the classification threshold.

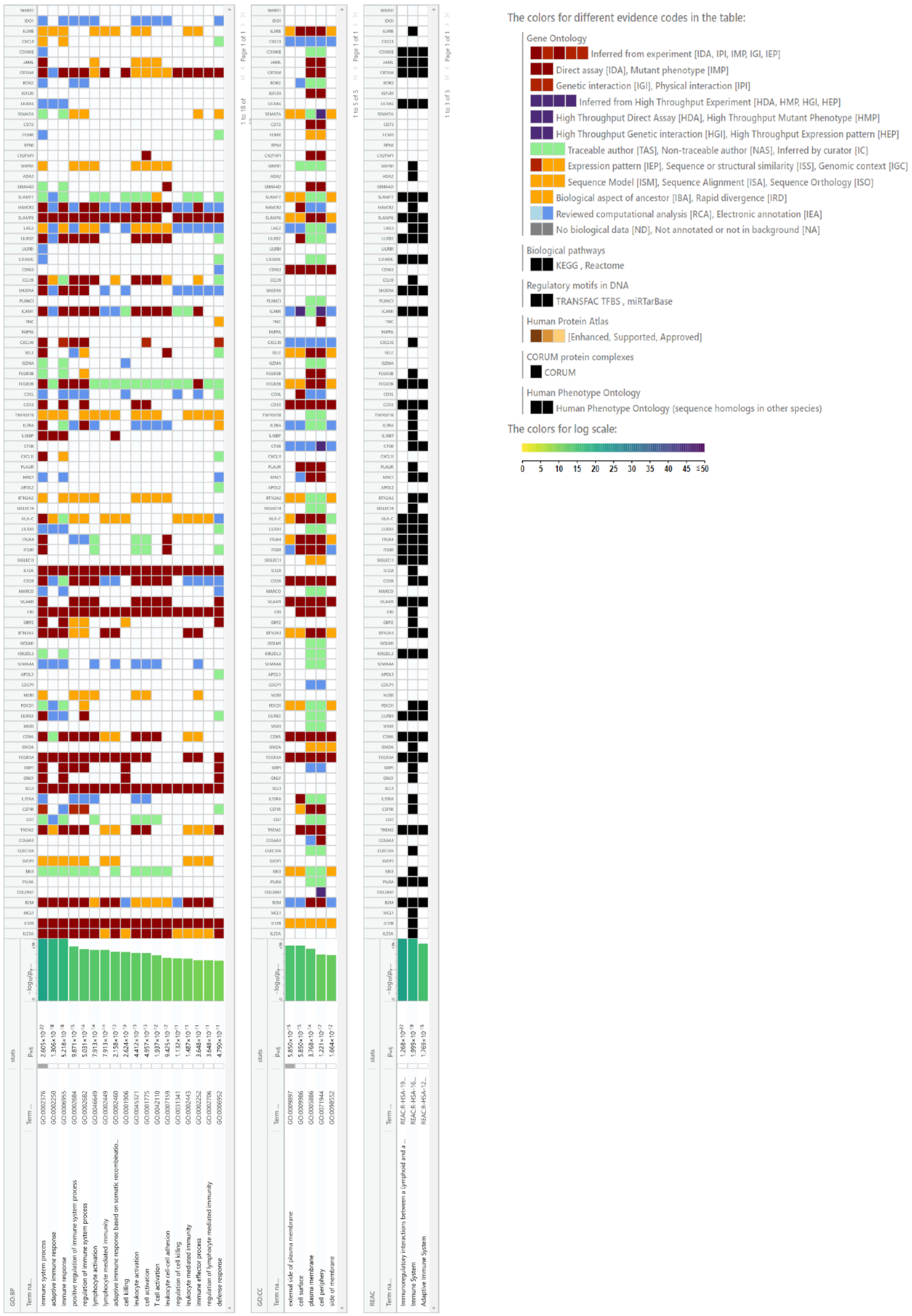

The colors for different evidence codes in the table:
Gene Ontology
Inferred from experiment [IDA, IPI, IMP, IGI, IEP]
Direct assay [IDA], Mutant phenotype [IMP]
Genetic interaction [IGI], Physical interaction [IPI]
Inferred from High Throughput Experiment [HDA, HMP, HGI, HEP]
High Throughput Direct Assay [HDA], High Throughput Mutant Phenotype [HMP]
High Throughput Genetic interaction [HGI], High Throughput Expression pattern [HEP]
Traceable author [TAS], Non-traceable author [NAS], Inferred by curator [IC]
Expression pattern [IEP], Sequence or structural similarity [ISS], Genomic context [IGC]
Sequence Model [ISM], Sequence Alignment [ISA], Sequence Orthology [ISO]
Biological aspect of ancestor [IBA], Rapid divergence [IRD]
Reviewed computational analysis [RCA], Electronic annotation [IEA]
No biological data [ND], Not annotated or not in background [NA]
Biological pathways
KEGG , Reactome
Regulatory motifs in DNA
TRANSFAC TFBS , miRTarBase
Human Protein Atlas
[Enhanced, Supported, Approved]
CORUM protein complexes
CORUM
Human Phenotype Ontology
Human Phenotype Ontology (sequence homologs in other species)
The colors for log scale:
0 5 10 15 20 25 30 35 40 45 ≤50
GO:BP
GO:CC
REAC

Figure S2: Enrichment of immune-related processes and membrane proteins. Enrichment analyses were performed on proteins exhibiting significant and positive fold changes (T1:T0) in the ICI monotherapy cohort (n=100). Top GO biological processes and GO cellular compartments are shown.

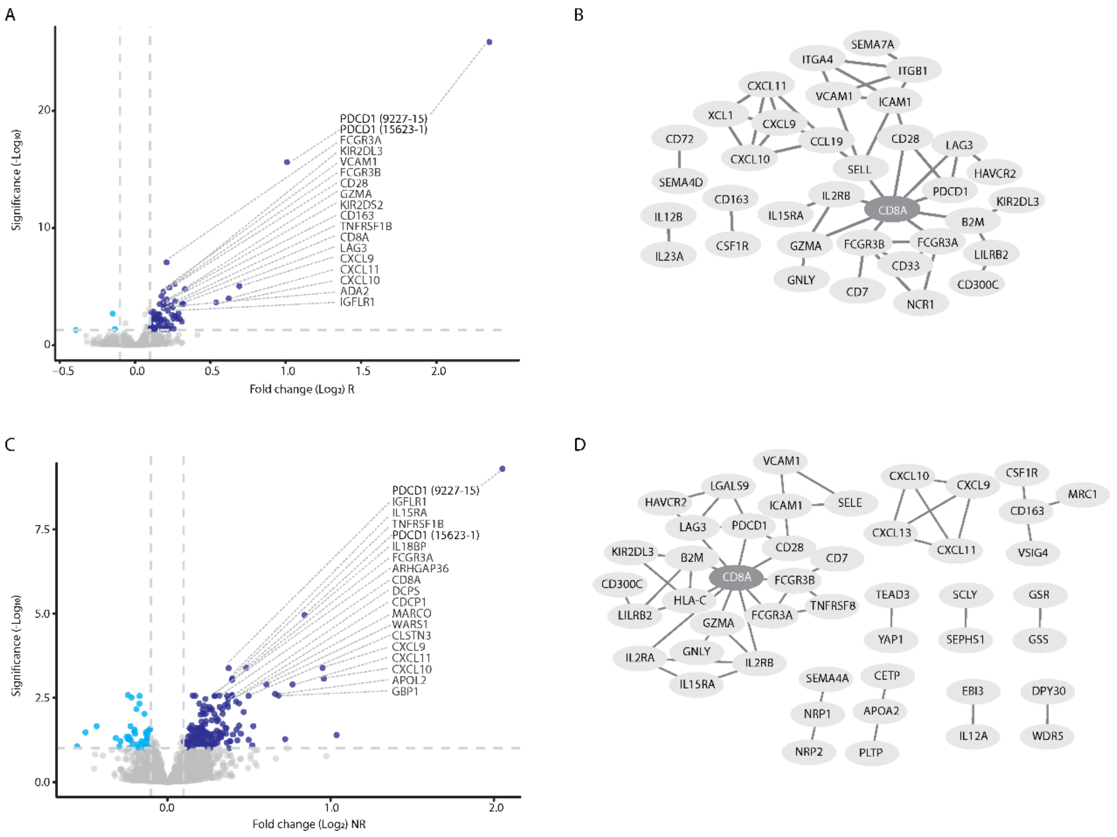

Figure S3: A T cell hub exists in responder and non-responder NSCLC patients, as defined by clinical benefit. Patients were stratified according to clinical benefit, such that the responder (R) cohort included patients with complete response, partial response or stable disease (n=71), and the non-responder (NR) cohort included patients with progressive disease (n=28). Shown are Volcano plots representing fold change in plasma protein levels (T1:T0) in patients treated with ICI monotherapy. R cohort is shown in (A) and NR cohort is shown in (C). Protein-protein interaction networks were generated from proteins with significant and positive fold change in R and NR protein sets, shown in (B) and (D), respectively. Interaction score with 0.9 confidence.

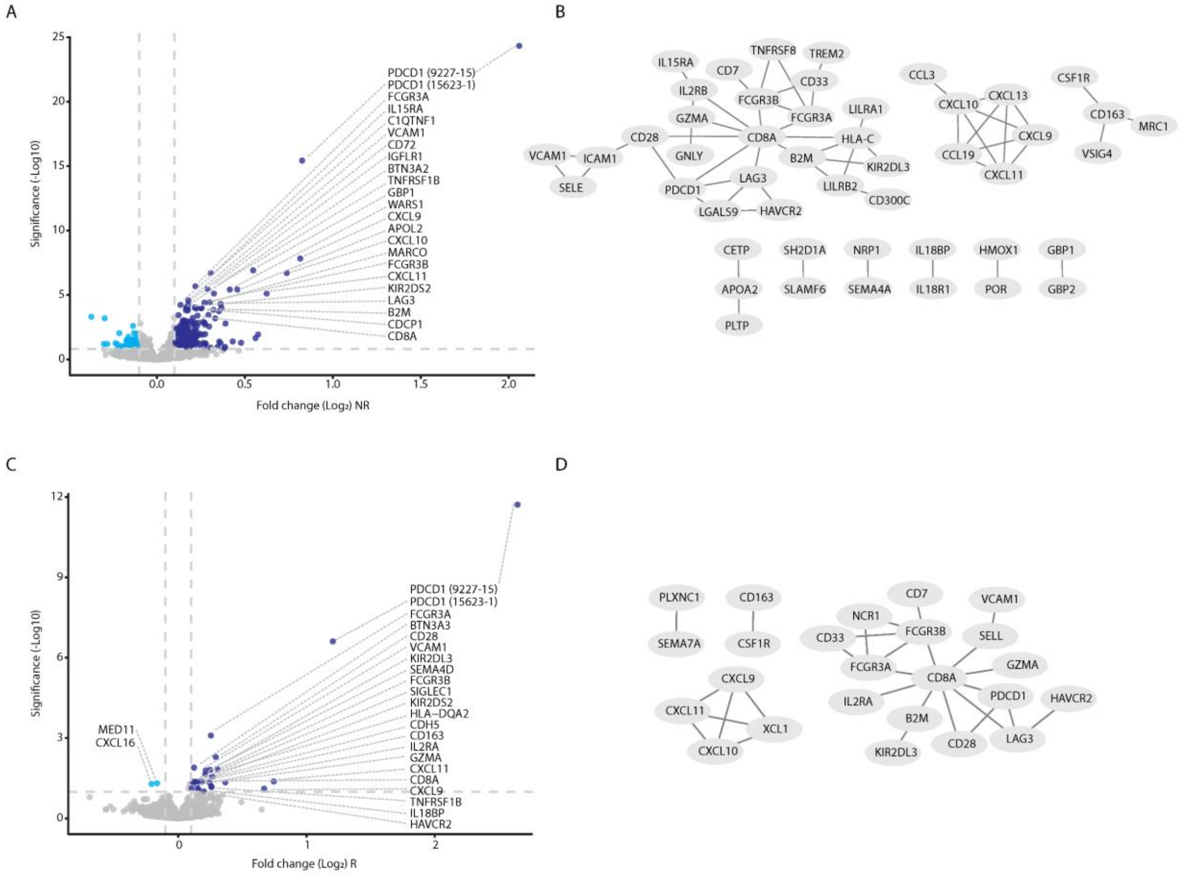

Figure S4: A T cell hub exists in responder and non-responder NSCLC patients, as defined by objective response. Patients were stratified according to objective response, such that the responder (R) cohort included patients with complete or partial response (n=36), and the non-responder (NR) cohort included patients with stable or progressive disease (n=63). Shown are Volcano plots representing fold change in plasma protein levels (T1:T0) in patients treated with ICI monotherapy. NR cohort is shown in (A) and R cohort is shown in (C). Protein-protein interaction networks were generated from proteins with significant and positive fold change in NR and R protein sets, shown in (B) and (D), respectively. Interaction score with 0.9 confidence

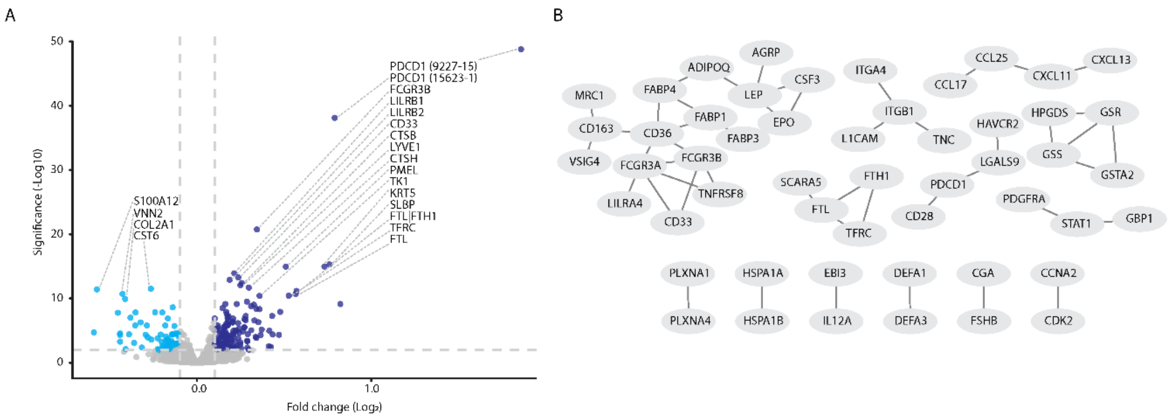

Figure S5: The addition of chemotherapy to ICIs weakens the T cell hub. A. Volcano plot representing fold change in plasma protein levels (T1:T0) in patients treated with PD-1/PD-L1 inhibitors combined with chemotherapy (n=125). B. A protein-protein interaction network was generated from proteins with significant and positive fold change. Interaction score with 0.9 confidence. Notably, protein-protein interactions do not present a T cell hub.

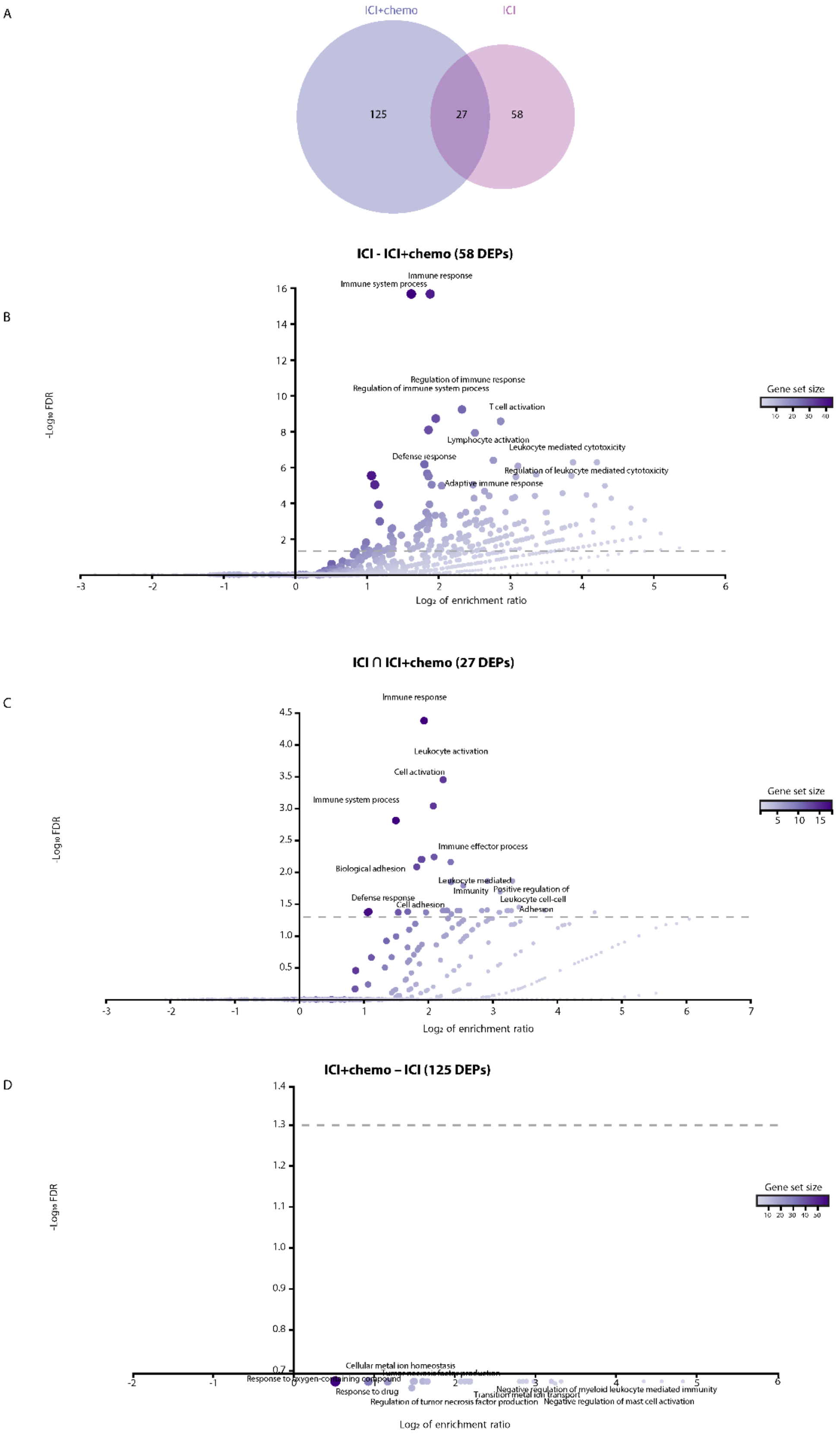
A
ICI+chemo
ICI
125
27
58
B
ICI - ICI+chemo (58 DEPs)
Immune response
Immune system process
Regulation of immune response
Regulation of immune system process
T cell activation
Lymphocyte activation
Leukocyte mediated cytotoxicity
Defense response
Regulation of leukocyte mediated cytotoxicity
Adaptive immune response
-Log₁₀ FDR
Log₂ of enrichment ratio
Gene set size
C
ICI ∩ ICI+chemo (27 DEPs)
Immune response
Leukocyte activation
Cell activation
Immune system process
Immune effector process
Biological adhesion
Leukocyte mediated
Immunity
Positive regulation of
Leukocyte cell-cell
Adhesion
Defense response
Cell adhesion
-Log₁₀ FDR
Log₂ of enrichment ratio
Gene set size
D
ICI+chemo – ICI (125 DEPs)
Cellular metal ion homeostasis
Tumor necrosis factor production
Response to oxygen-containing compound
Response to drug
Regulation of tumor necrosis factor production
Transition metal ion transport
Negative regulation of myeloid leukocyte mediated immunity
Negative regulation of mast cell activation
-Log₁₀ FDR
Log₂ of enrichment ratio
Gene set size

Figure S6: T cell-related processes are highly enriched in the ICI monotherapy cohort. A. Venn diagram showing number of proteins with significant and positive fold changes (T1:T0) in ICI monotherapy (ICI) and ICI combined with chemotherapy (ICI+chemo) cohorts. B-D. Pathway enrichment analyses were performed on the following protein sets: 58 proteins exclusive to ICI cohort (B); 27 proteins found in both cohorts (C); 125 proteins exclusive to ICI+chemo cohort (D). The analysis was performed using WebGestalt.

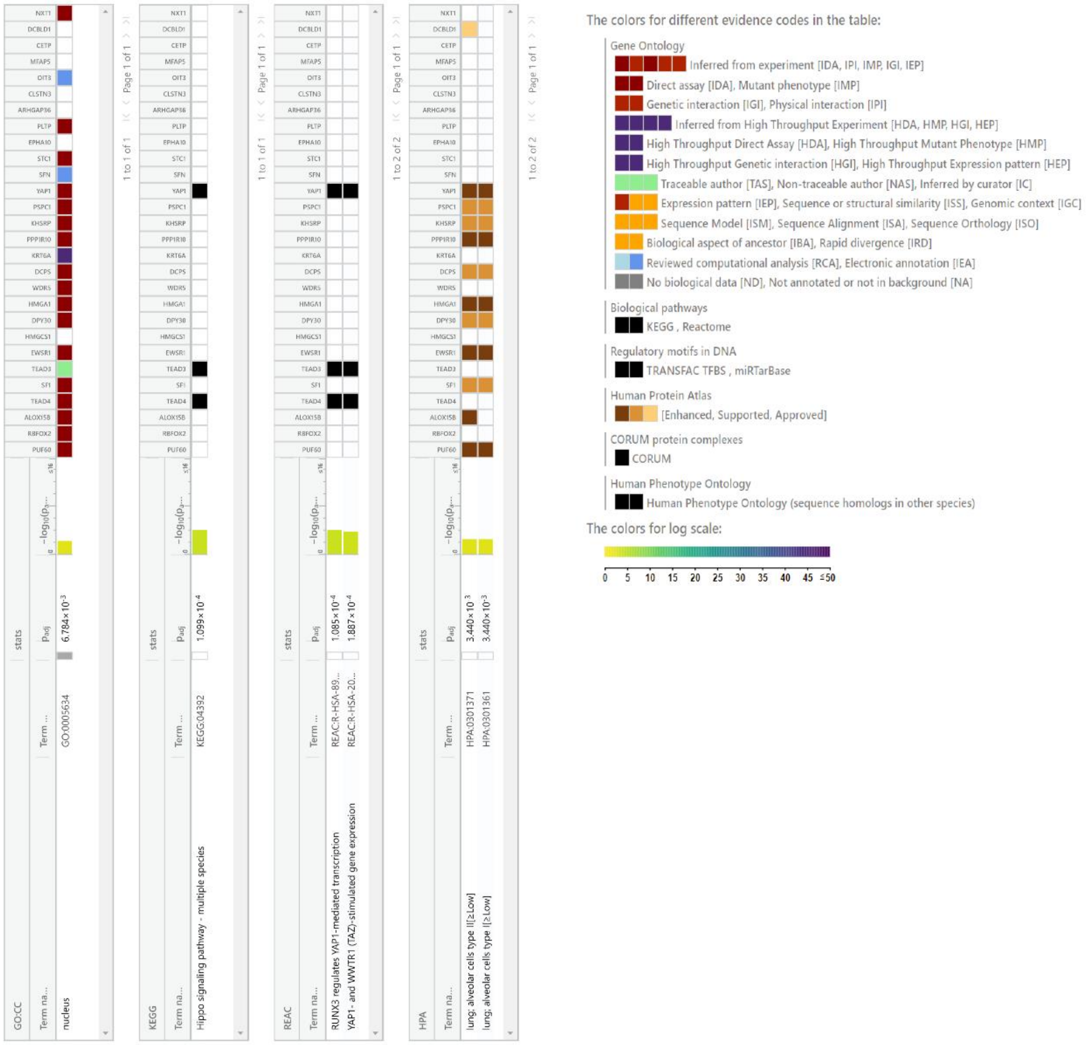

Figure S7: Nuclear or intracellular proteins are enriched in non-responder NSCLC patients. Enrichment analyses were performed on proteins exhibiting significant and positive fold changes (T1:T0) in the non-responder subcohort of NSCLC patients treated with ICI monotherapy (n=27). Top results from GO cellular compartments and Human Protein Atlas are shown.

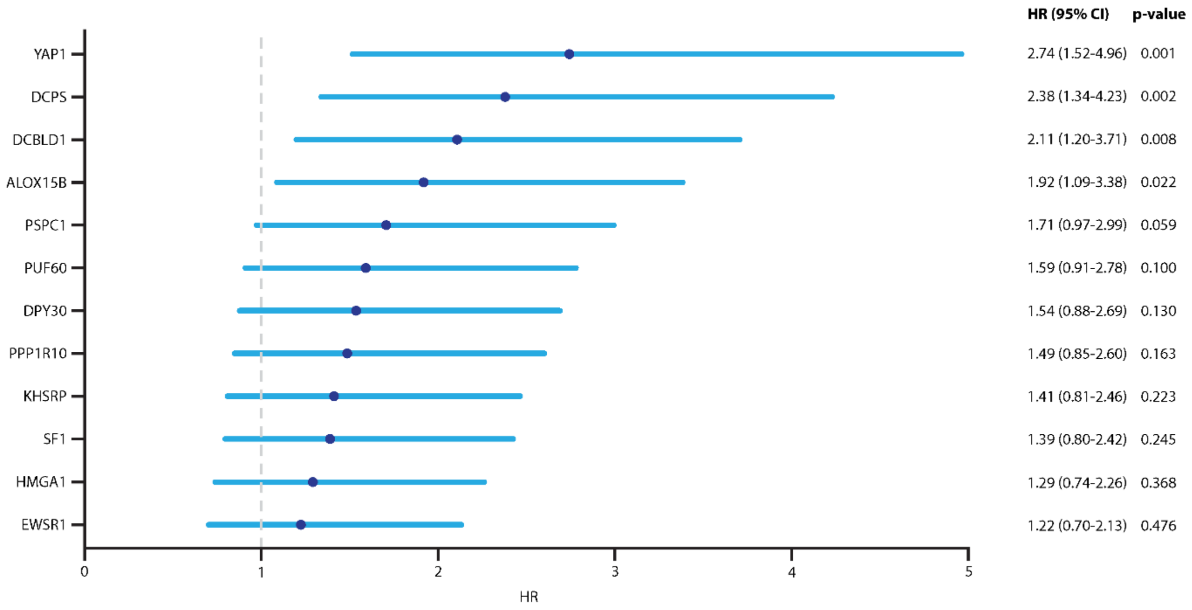

Figure S8: Kaplan-Meier hazard ratios showing the relationship between selected alveolar proteins and overall survival. The analysis was performed on the ICI monotherapy patient cohort (n=100). Patients were classified as having high or low fold change for each of the indicated alveolar proteins, using the median fold change as the classification threshold. Hazard ratios (HR) are shown.

A

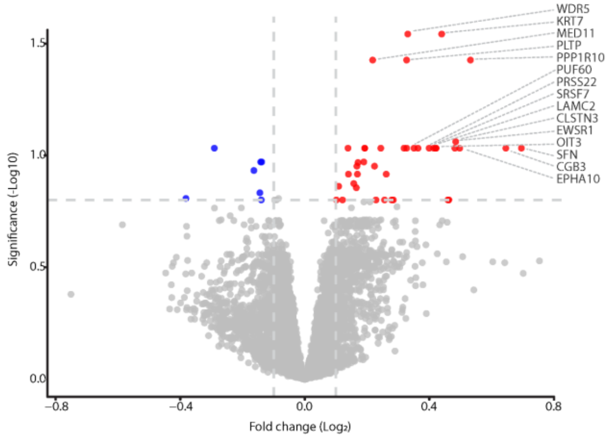

B

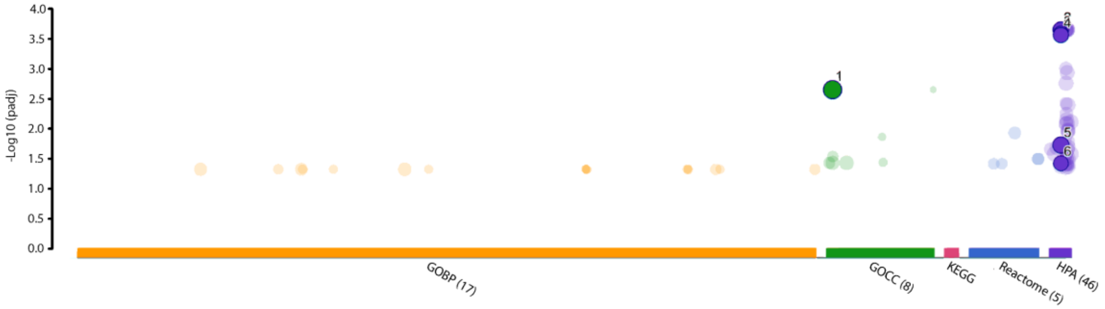

ID	Sour...	Term ...	Term Na...	p_{adj} (query...
1	GO:CC	GO:0005634	nucleus	2.264×10^{-3}
2	HPA	HPA:0301361	lung; alveolar cells type I[≥Low]	2.247×10^{-4}
3	HPA	HPA:0301363	lung; alveolar cells type I[High]	2.533×10^{-4}
4	HPA	HPA:0301362	lung; alveolar cells type I[≥Medium]	2.754×10^{-4}
5	HPA	HPA:0301371	lung; alveolar cells type II[≥Low]	1.903×10^{-2}
6	HPA	HPA:0301373	lung; alveolar cells type II[High]	3.838×10^{-2}

Figure S9: Intracellular alveolar-associated proteins are enriched in plasma of non-responder NSCLC patients, as defined by objective response. Patients were stratified according to objective response, such that the responder (R) cohort included patients with complete or partial response (n=36), and the non-responder (NR) cohort included patients with stable or progressive disease (n=63). A. Volcano plot representing fold change in plasma protein levels [T1:T0 fold change in non-responders (NR) vs T1:T0 fold change in responders (R)] in patients treated with ICI monotherapy. B. Pathway enrichment analysis of proteins exhibiting significant and positive fold change (FC>0.1 and q values<0.15). Categorization is based on gProfilier.

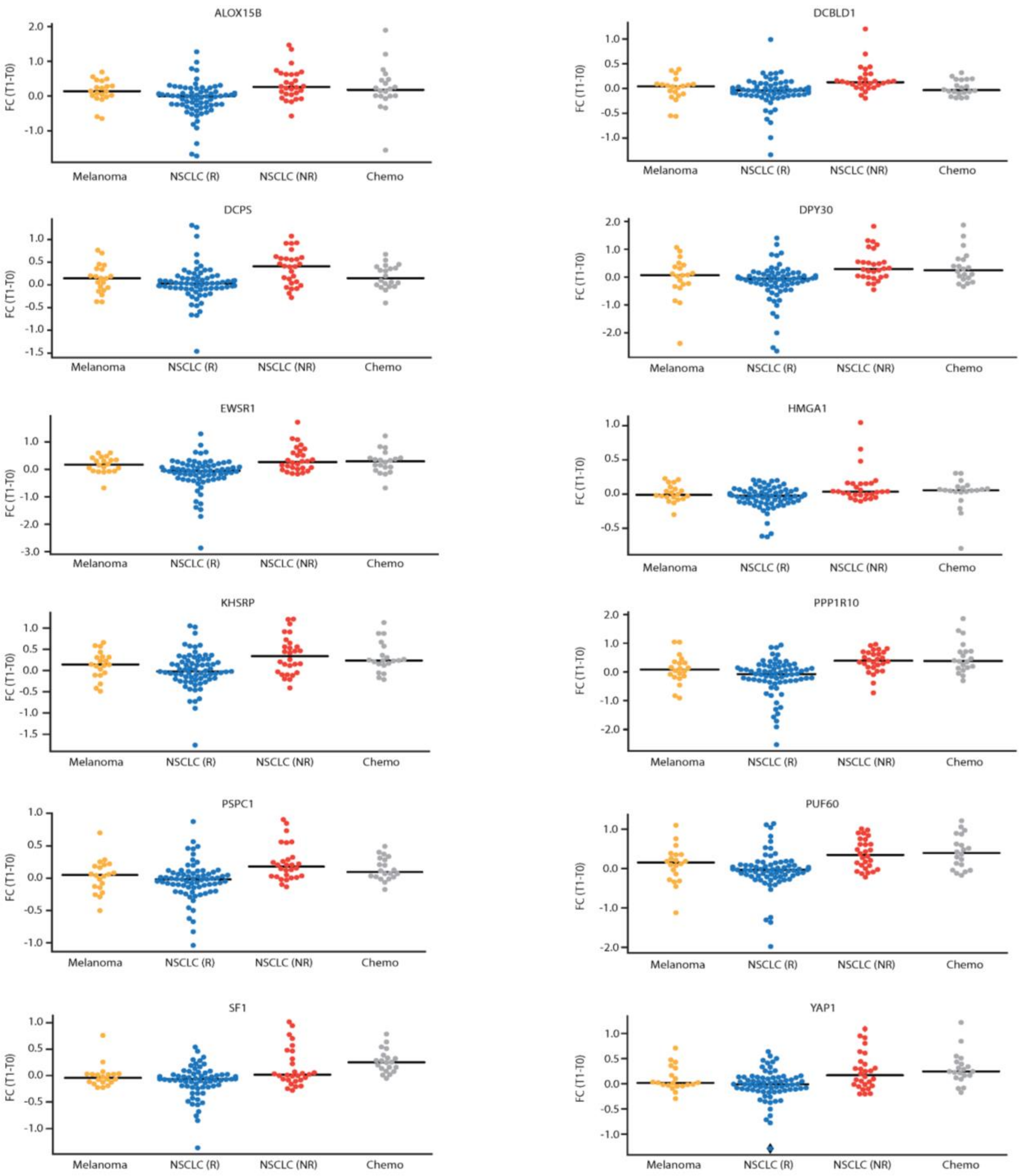

Figure S10: Fold change of selected alveolar proteins in different patient cohorts. Proteins (with potential alveolar origin) displaying significantly higher plasma levels in non-responder (NR) compared to responder (R) NSCLC patients treated with ICI monotherapy were selected for analysis in different patient cohorts. Shown are the fold changes (T1:T0) per protein in R and NR NSCLC patients treated with ICI monotherapy, NSCLC patients treated with chemotherapy and melanoma patients treated with ICI-based therapy.
